# Versatile Virus-Like Particle Carrier for Epitope Based Vaccines

**DOI:** 10.1371/journal.pone.0009809

**Published:** 2010-03-23

**Authors:** Alain C. Tissot, Regina Renhofa, Nicole Schmitz, Indulis Cielens, Edwin Meijerink, Velta Ose, Gary T. Jennings, Philippe Saudan, Paul Pumpens, Martin F. Bachmann

**Affiliations:** 1 Cytos Biotechnology AG, Zurich-Schlieren, Switzerland; 2 Latvian Biomedical Research and Study Centre, Riga, Latvia; Instituto Butantan, Brazil

## Abstract

**Background:**

Recombinant proteins and in particular single domains or peptides are often poorly immunogenic unless conjugated to a carrier protein. Virus-like-particles are a very efficient means to confer high immunogenicity to antigens. We report here the development of virus-like-particles (VLPs) derived from the RNA bacteriophage AP205 for epitope-based vaccines.

**Methodology/Principal Findings:**

Peptides of angiotensin II, *S.typhi* outer membrane protein (D2), CXCR4 receptor, HIV1 Nef, gonadotropin releasing hormone (GnRH), *Influenza A* M2-protein were fused to either N- or C-terminus of AP205 coat protein. The A205-peptide fusions assembled into VLPs, and peptides displayed on the VLP were highly immunogenic in mice. GnRH fused to the C-terminus of AP205 induced a strong antibody response that inhibited GnRH function *in vivo*. Exposure of the M2-protein peptide at the N-terminus of AP205 resulted in a strong M2-specific antibody response upon immunization, protecting 100% of mice from a lethal influenza infection.

**Conclusions/Significance:**

AP205 VLPs are therefore a very efficient and new vaccine system, suitable for complex and long epitopes, of up to at least 55 amino acid residues in length. AP205 VLPs confer a high immunogenicity to displayed epitopes, as shown by inhibition of endogenous GnRH and protective immunity against influenza infection.

## Introduction

Vaccination has been one of the most effective ways to control pathogens and prevent disease in the history of medicine. Essentially all successful prophylactic vaccines work by inducing neutralizing or protective antibodies against pathogens (polio, small pox, tetanus) [1]–[3]. The first attempts to vaccinate were dominated by live vaccines as e.g., vaccinating against small pox using first small pox virus itself followed by vaccinia virus. A constant search for safer vaccines has led to the development of live attenuated (e.g. Polio Sabin), inactivated (e.g. Polio Salk) and finally recombinant vaccines based for example on subunits of viral surface proteins (e.g. HBV and HPV vaccine) [2], [4], [5]. Regulatory authorities and also public opinion ask for ever safer and better characterized vaccines [6]. In this respect, the use of a small portion of an antigen, such as an epitope, instead of full-length proteins represents a potentially safer vaccine, since it limits the scope of potential cross-reactivity and concentrates the antibody response against the desired portion of an antigen. This is particularly advantageous when targeting self-antigens, as is the case for immunotherapeutics, which represent a new promising class of vaccines developed to treat chronic diseases [7]–[9].

Many epitopes are short peptides, which immunogenicity is low unless conjugated to a carrier protein. Currently, there is a limited set of carrier proteins used to generate most of such conjugate vaccines. One of them is the ubiquitously used Keyhole limpet hemocyanin (KLH), and two other carrier proteins are toxoids which are themselves component of other vaccines, Tetanus toxoid (tetanus vaccine) or Diptheria toxoid (diphteria vaccine).

Due to the complexity of conjugate vaccine formation, which requires production of the carrier protein, epitopes as well as chemical conjugation, the cost of goods may sometimes be prohibitive [8]. A simpler mean to produce such conjugate vaccines may be to genetically rather than chemically link the peptide epitopes to the carrier protein. However, the insertion of epitopes into carrier proteins and in particular virus-like particles (VLPs), remains fairly unpredictable despite some remarkable successes [10]–[13]. Major limitations include accessibility of the epitope, size limitations, perturbation of the structure of the viral protein by the inserted epitope, and enforcement of a non-native structure on the epitope. In addition, some of the viral carriers face major challenges for regulatory approval due to infectivity or presence of DNA and resistance genes.

Recently, we have described new vaccines based on a VLP derived from the RNA bacteriophage Qβ, made from 180 subunits assembling into an icosahedral capsid in *E. coli*. Epitopes conjugated chemically to these VLPs are displayed in a highly repetitive and organized manner, inducing strong immune responses in all species tested [14]. Remarkably, the highly repetitive display allows to elicit strong antibody responses even against self-antigens [15]–[20]. Indeed, vaccines based on epitopes coupled to Qβ have shown clinical efficacy in smoking cessation and the treatment of hypertension [21]–[24].

In this report, we describe a new RNA phage based VLP-vaccine carrier allowing both N- or C-terminal fusion of epitopes. The VLP is formed from 180 copies of the coat protein. We show that long (up to at least 55 amino acids) or complex epitopes (containing multiple cysteine residues) can be fused to this AP205-derived VLP-carrier. One vaccine successfully induced antibodies against GnRH, a self-antigen involved in fertility and prostate cancer. A second epitope fused to AP205 VLPs was the extracellular domain of the *Influenza A* M2 protein. Vaccination of mice with M2-AP205 resulted in 100% protection from lethal infection with influenza virus.

## Results

**Figure 1:**
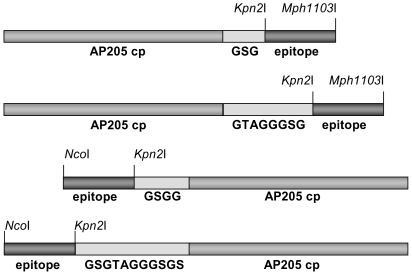
Schematic representation of the four AP205 coat protein fusion constructs. Genes encoding an epitope to be introduced at the N-terminus of AP205 using a short spacer (in plasmid pAP378) or a long spacer (in plasmid pAP382) are cloned as *Nco*I-*Kpn*2I cassettes, while for introduction of epitopes to the C-terminus of AP205 coat protein using a short spacer (in plasmid pAP409) or a long spacer (in plasmid pAP405) a *Kpn*2I- *Mph1103*I cassette is used. AP205 cp stands for AP205 coat protein sequence.

**Figure 2:**
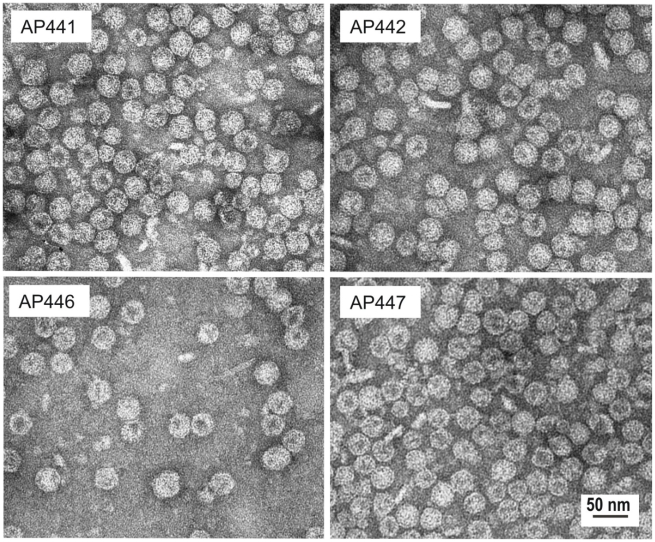
Electron micrographs of negatively stained AP205-Ang II VLPs. AP205 VLPs displaying the Ang II peptide fused to the C-terminus (AP441 – short linker, AP442 – long linker), or the N-terminus (AP446 – short linker, AP447 – long linker) of AP205 coat protein.

**Figure 3:**
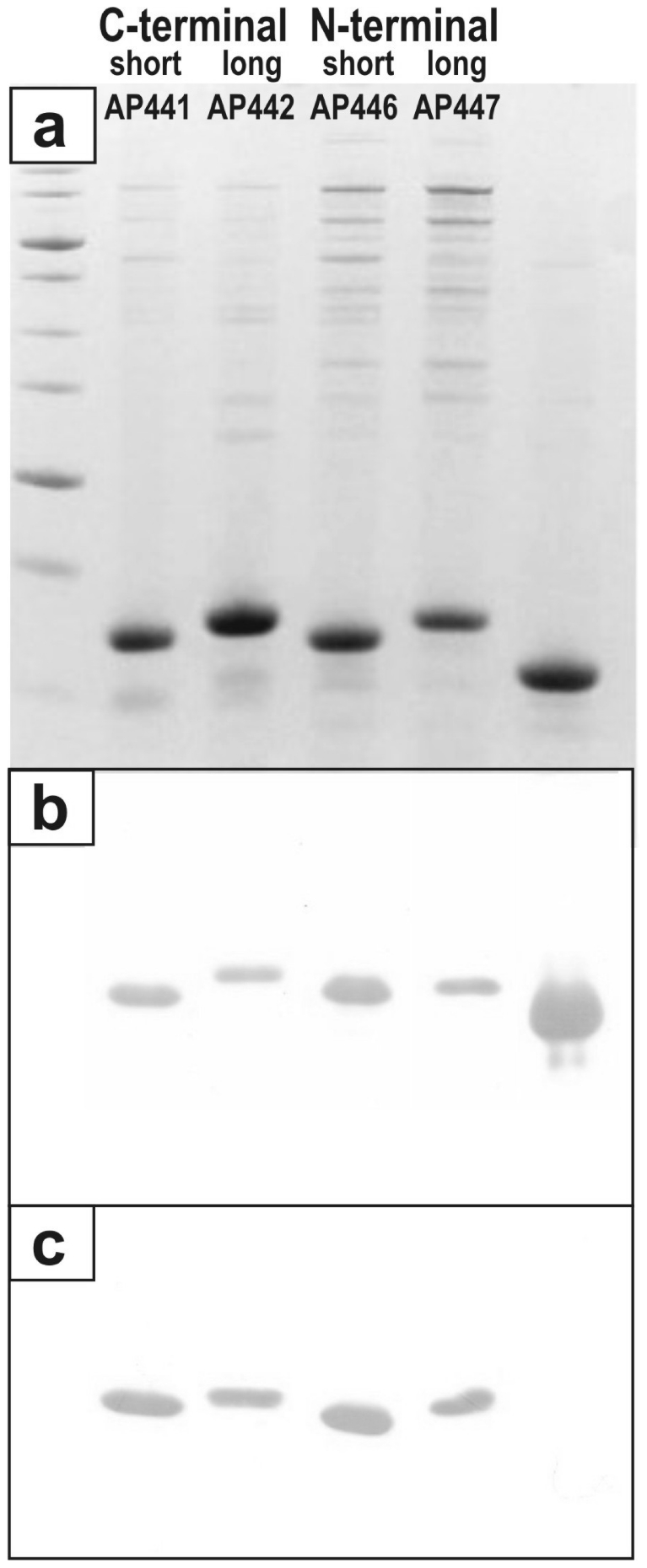
AP205-Ang II VLPs analyzed by dodecyl sulphate PAGE and Western blot. After purification by gel filtration, AP205 VLPs carrying Ang II peptide fused to the C-terminus (AP441 and AP442) or N-terminus (AP446 and AP447) of AP205 coat protein were heated at 95°C in sample buffer for 5 minutes prior to loading. (a) Coomassie-stained LDS-PAGE of corresponding chimeric proteins; (b) Western blot with an AP205 coat protein-specific rabbit serum; (c) Western blot with an Ang II-specific mouse serum. The migration of all four AP205 coat proteins carrying Ang II is retarded in comparison to wt AP205 coat protein (130 aa). Accordingly, migration of coat protein fused to AP205 coat protein using a long spacer (AP442 and AP447) is slower than the migration of coat proteins containing a short spacer (AP441 and AP446).

**Figure 4:**
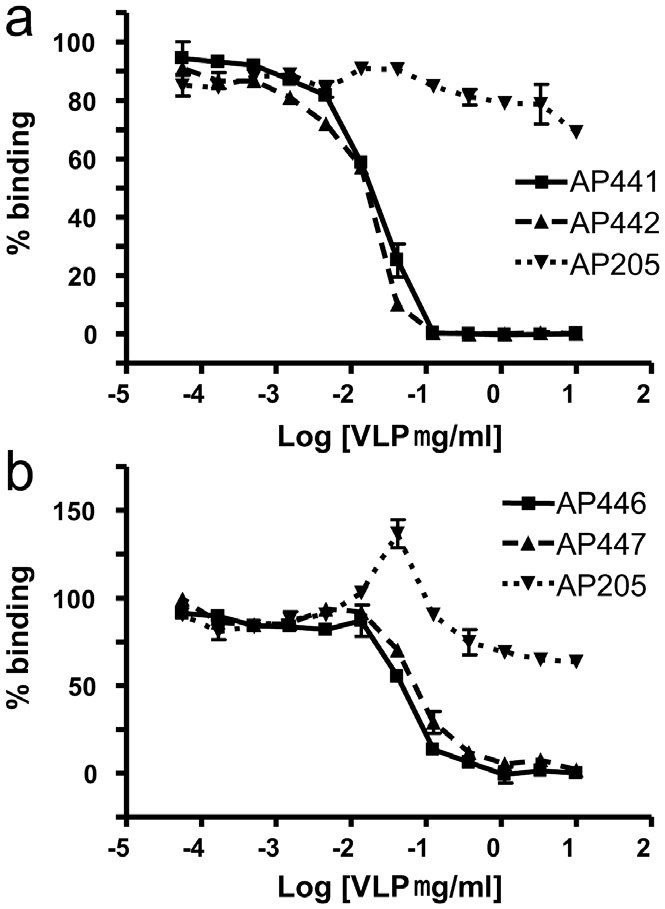
Display of Ang II on AP205 VLP assessed by inhibition ELISA. Ang II was conjugated to RNAse, and coated on ELISA plates. The anti-Ang II serum was pre-incubated with serial dilutions of the 4 VLPs displaying Ang II or wt AP205, and subsequently added to the wells coated with RNAse-Ang II. Signals shown on the Figure are normalized and are given as % binding. The VLP concentration [µg/ml] was log transformed. (a) VLPs displaying Ang II at the C-terminus of AP205 coat protein using a short (AP441) or long spacer (AP442) or wt AP205 VLP (AP205). (b) VLPs displaying Ang II at the N-terminus of AP205 coat protein using a short (AP446) or long spacer (AP447) or wt AP205 VLP (AP205).

AP205 has been recently identified as a new RNA bacteriophage infecting the Gram-negative bacteria *Acinetobacter sp*. Sequence alignment of its coat protein with other RNA phage coat proteins is difficult since only 5 amino acids (aa) are conserved among all RNA phages isolated so far, even when using structural information [25], [26]. The N- and C-termini of RNA bacteriophage coat proteins come close together at the three-fold axis of their icosahedral shell and are often involved in interactions with each other, or shielded from the surface. We generated four AP205 genes, allowing rapid insertion of oligonucleotides or genes encoding a desired epitope to both the N- and C-terminus of AP205 coat protein, using either a short (3 or 4) or long (8 or 11) aa spacer to ensure optimal display of the epitope (Fig. 1). Whilst the shorter spacers were combinations of G and S residues, the long spacer sequences used were modifications of a linker sequence present in the so called A1 extension of Qβ coat protein [27]. Table 1 presents a list of epitopes vs. VLP vector and spacer variants used for the construction of model vaccines. In a first set of experiments, a short peptide, angiotensin II (Ang II), was fused to the N- and C-terminus of the coat protein, using all four vectors. The resulting fusion proteins were expressed in *E. coli*, and spontaneously assembled into VLPs as detected by electron microscopy (Fig. 2). VLPs were purified by gel filtration and tested by Coomassie staining after LDS-PAGE (Fig. 3a). Western blot analysis of the fused proteins using an antiserum against AP205 (Fig. 3b) or Ang II (Fig. 3c) confirmed presence of the angiotensin peptide. In the absence of a crystal structure of AP205 VLP (Tars et al., in progress), the accessibility of epitopes fused at any one of the coat protein termini can only be tested experimentally. To this end, we assessed the ability of the various AP205 VLPs displaying Ang II to inhibit binding of an Ang II-specific antiserum to an Ang II-conjugate coated on an ELISA plate (Fig. 4a and b). VLPs were preincubated with the antiserum specific for Ang II and remaining free antibodies were detected by ELISA. As seen in Fig. 4a and 4b, both the VLPs with Ang II fused to the C-terminus, or the N-terminus of AP205 coat protein, inhibited binding of the anti-Ang II antiserum. Wild type AP205 VLP did not inhibit antibody binding, confirming the specificity of the inhibition and thereby the accessibility of the peptides on the surface of the VLPs.

**Table 1:** Epitopes and AP205 VLPs referred to in the manuscript.

Epitope			C-terminal fusions		N-terminal fusions	
Designation	Source	Length, aa	short linker	long linker	short linker	long linker
Ang II	Angiotensin II, aa 1–8	8	**+**	**+**	**+**	**+**
D2	*S.typhi* outer membrane protein, aa 266–280	15	**+**	**+**	**+**	**+**
CXCR4	CXCR4, N-terminal extracellular part, aa 1–39	39	−	−	**+**	−
Nef55	HIV Nef, aa 66–100, 132–151	55	−	**+**	−	−
GnRH	Gonadotropin releasing hormone, aa 1–10	10	−	**+**	−	−
M2	M2, N-terminal ectodomain, aa 2–24	24	−	**−**	**+**	−

AP205 VLPs are designated by the linker used to fuse the corresponding epitope. Combinations described in the manuscript are referred to with a “+”.

**Figure 5:**
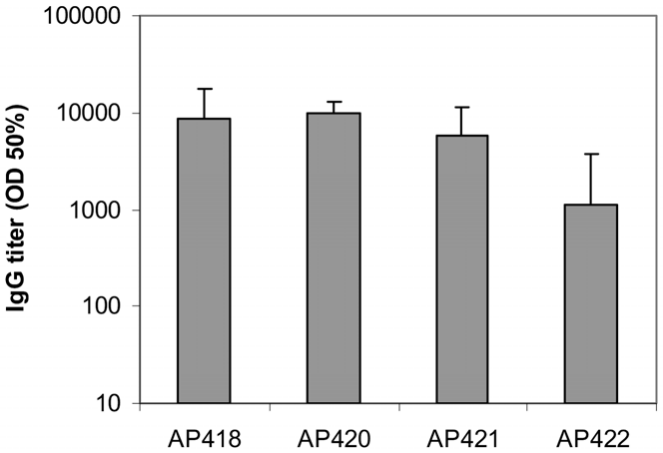
Immunogenicity of D2 peptide displayed on AP205 VLPs. AP205 VLPs with the D2 peptide fused to the C-terminus (AP418, short linker, and AP420, long linker) and to the N-terminus (AP421, short linker, and AP422, long linker) were injected s.c. at a dose of 25 µg twice, on day 0 and 14. Sera were collected on day 21, and IgG titre measured by ELISA. Pre-immune titre was too low to be determined, and was arbitrarily attributed the value of 1∶100, the lowest dilution of serum used in the assay.

The display of the fused epitopes on the surface of the AP205-VLP would predict that these epitopes should be highly immunogenic. In order to test immunogenicity and at the same time to assess whether AP205 coat protein could accommodate epitopes larger than the 8 aa peptide Ang II, we fused a sequence encoding the *Salmonella typhi* outer membrane protein derived 15 aa peptide D2 [28] to AP205 coat protein, using our four vectors. The resulting coat proteins were expressed in *E.coli* and the VLPs purified. All four coat-protein fusions assembled into VLPs, as was tested by size exclusion chromatography (not shown) and electron microscopy (Fig. S1). After purification of chimeric AP205-D2 VLPs by gel filtration to reliable purity confirmed by LDS-PAGE (Fig. S2), competitive ELISA showed that the D2 peptides are displayed on the outer VLP surface to the same extent as the Ang II peptide in the corresponding VLPs (not shown). In order to further test accessibility of the peptide on the VLP and to test the immunogenicity of the particles, we immunized Balb/c mice at fortnightly interval with 25 µg of D2-VLPs in the absence of adjuvant. As shown in Fig. 5, all 4 VLPs were highly immunogenic, and a robust titer was measured after two injections. The response is specific for the D2 peptide, as AP205 linked to a control peptide induces antibodies that do not bind to D2 (data not shown). Display of the D2 peptide with a long linker at the N-terminus of AP205 VLP seemed, however, to yield less immunogenic VLPs than using a short linker at the N-terminus, or displaying the peptide at the C-terminus of the VLP carrier. These data independently confirm the accessibility of fused epitopes on the surface of AP205 VLP.

In order to investigate whether peptides of greater length can be displayed on AP205 VLP, we fused the N-terminal 39 aa extracellular part of CXCR4 to the N-terminus, and a 55 aa HIV polyepitope containing Nef consensus T-cell epitopes to the C-terminus of AP205 VLP. Both fusion coat proteins assembled into VLPs and displayed their epitopes, as was confirmed by electron microscopy (Fig. S3), SDS-PAGE and Western blot (not shown). Thus, peptides as large as 55 aa in length can be fused to AP205 VLP.

We wanted next to see whether the high immunogenicity of the AP205-VLP vaccines could generate antibodies neutralizing their target and having a useful therapeutic effect *in vivo*. To this end, we selected GnRH as a target for neutralisation in mice. This provided additionally the opportunity to test whether AP205 VLP based vaccines could break B cell unresponsiveness towards self-antigens. This is important, as a number of attractive molecules currently targeted for immunotherapy are self-antigens. Antibodies neutralising GnRH have the potential to act as androgen-deprivation therapy used to date to treat prostate cancer [29]–[31], since prostate cancer cells depend on testosterone for growth [32]. Fertility management may be an additional potential use of such a vaccine [33]. Therapeutically meaningful induction of neutralising antibodies against GnRH should lead to a decrease in LH and FSH production and subsequently to reduced levels of androstenone and gonadal steroids like estrogens and testosterone. This in turn will cause testis atrophy that can be monitored as testis weight reduction.

**Figure 6:**
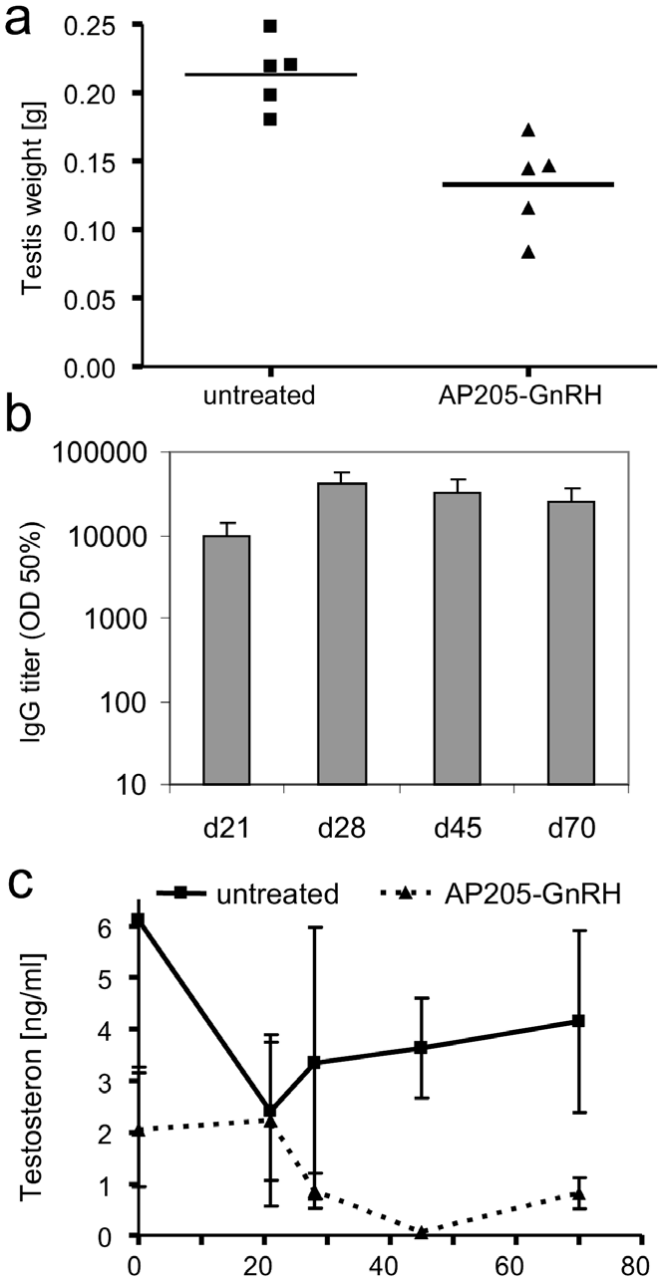
Inhibition of GnRH function in mice immunized with AP205-GnRH. (a) Testis weight in male C57BL/6 mice immunized with 50 µg AP205 GnRH, or untreated, on day 0 and 21. Mice were sacrificed on day 70. There was a 38% decrease in testis weight in the AP205-GnRH group compared to the untreated control group (p = 0.003, *t*-test). (b) Anti-GnRH IgG antibody titre measured by ELISA. The mice were immunized on day 0, and 21 with 50 µg AP205-GnRH, and anti-GnRH ELISA titres measured on day 21, 28, 45, and 70. (c) Testosterone level measurements. Testosterone levels were measured by ELISA in sera collected on day 0, 21, 28, 45 and 70. Error bars are the standard error of the mean. The median of the testosterone level, averaged between day 28 and 70, was 81% lower in the AP205-GnRH treated animals when compared to untreated animals (p = 0.008, Mann-Whitney test).

We therefore fused murine GnRH to the C-terminus of AP205 coat protein, using a long spacer. The fused coat protein assembled to VLPs in *E.* coli as determined by electron microscopy of purified VLPs. Presence of the peptide in AP205-VLPs was confirmed by western blot. Male adult mice were immunized once at 9 weeks of age with 50 µg of AP205-GnRH and boosted three weeks later with the same dose. Neutralization of GnRH activity was assessed by measuring testis weight 70 days after initial immunisation. As seen in Fig. 6a, immunisation with AP205-GnRH led to a 38% decrease in testis weight (p = 0.003, *t*-test), demonstrating the neutralization activity of raised antibodies. Antibody titers and testosterone levels were monitored on day 0, 21, 28, 45 and 70. A high antibody titer was already achieved after the first immunisation in all animals on day 21, and was further boosted after the second injection (Fig. 6b), further confirming that the immunisation had efficiently overcome B-cell unresponsiveness. The response against GnRH is specific, as antibodies generated against AP205 alone do not recognize GnRH (data not shown). As expected, GnRH neutralization led to a decrease in testosterone levels, and we observed a reduction in testosterone starting from day 28, coincidentally with reaching high antibody titers after the booster immunisation (Fig. 6, b and c). The median of the testosterone level, averaged between day 28 and 70, was 81% lower in the AP205-GnRH treated animals when compared to untreated animals (p = 0.008, Mann-Whitney test).

**Figure 7:**
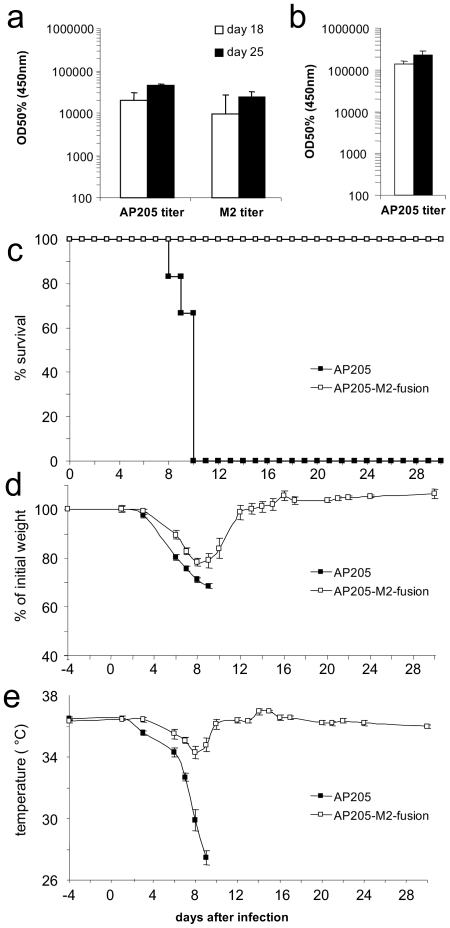
Protection of mice by immunization with M2-AP205 against a lethal influenza challenge. Mice were immunized on day 0 and 18 with M2-AP205 or AP205, and challenged with 4× the lethal dose of influenza Pr8 (H1N1) on day 31. (a) Anti-M2 and anti-AP205 IgG titre on day 18 and 25 in mice immunized with M2-AP205. (b) Anti-AP205 IgG titer on day 18 and 25 in mice immunized with AP205 only. (c) Survival curve. 4 control animals had to be sacrificed on day 10 due to excessive weight loss and temperature drop. Morbidity was assessed by measurement of the animal weights (d) and temperature drop (e).

The experiments above demonstrate that AP205 VLP induced rapidly a high antibody response in all animals after a single injection. This is particularly advantageous in cases where a rapid immune response has to be mounted against a pathogen, for example in the case of an influenza pandemic. Currently, influenza vaccines contain as antigens hemagglutinin and neuraminidase from three strains selected every year by the WHO. Use of a conserved antigen not subject to yearly variation would be a considerable improvement. The N-terminal M2 epitope of influenza is such a conserved epitope [34]–[36]. Antibodies raised against M2 do not neutralise influenza virions, but rather act through antibody dependent cytotoxicity (ADCC) to limit and clear viral infection [35]. In order to test whether an AP205 VLP based vaccine may elicit a high enough antibody responses to protect against influenza virus-infection, we fused the N-terminal ectodomain of *Influenza A* M2 protein to the N-terminus of AP205 VLP using a short spacer. The coat proteins fused to M2 assembled into VLPs, as was assessed by electron microscopy. The protective effect of the M2-AP205 VLPs against a challenge with the influenza PR 8 strain was assessed in C57BL/6 mice. The animals were immunized twice with M2-AP205 or AP205 only, and challenged intranasally (i.n.) 13 days after the last injection with a lethal dose of influenza virus. The mice mounted a strong immune response against M2 as well as against AP205 already after the first injection, and this response was boosted after the second injection (Fig. 7a). The mice immunized with control AP205 produced high antibody titers against AP205 but not against M2. All mice immunized with M2-AP205 were protected against the influenza challenge, while for the control group, all mice succumbed to the lethal infection (Fig. 7b). While control animals experienced severe weight and temperature loss M2-AP205 vaccinated animals were largely protected from symptoms of disease.

## Discussion

VLPs are very potent inducers of antibody responses not only against themselves but also against essentially any antigen displayed on them. Here we describe a new RNA-phage derived VLP that allows genetic fusion of peptide epitopes for display on their surface. Using various antigens, including a self-peptide, we demonstrate that this VLP-epitope display platform is able to induce potent antibody responses neutralizing GnRH in vivo and protecting mice from lethal infection with influenza virus. Moreover, the antibody response generated with AP205 VLPs is largely comparable in magnitude and IgG-subtype pattern (Th1) to what is obtained using RNA phage Qβ (unpublished), whose immunogenicity has been demonstrated in humans [22]–[24].

Remarkably, these AP205-VLPs were very receptive in accepting foreign sequences, and self-assembled even when an epitope with multiple cysteines such as the M2 epitope was fused to the coat protein. The longest epitope we have fused so far to the N-terminus of AP205 coat protein was 39 aa long, while to the C-terminus it was 55 aa in length. Chemical peptide synthesis becomes challenging with peptide length beyond 40 aa and this new technology therefore widens the range of epitopes available for vaccination. The ability to fuse to both ends of the AP205 VLP-subunit allows better display of N-or C- terminal epitopes which is often difficult for other VLPs. Indeed, VLPs such as those derived from the Hepatitis B core antigen (HBcAg), the bacteriophage fr or filamentous phage particles only allow fusion at one terminus of the subunit [37], [38]. The availability of VLPs suitable for fusing epitopes at either end is important, since for a number of epitopes, the protective immune response raised, is specific for their N- or C-terminal part (unpublished and [21]). In addition, this will allow to more exhaustively test epitopes when screening a whole pathogen genome or “antigenome” for antigenicity as recently described [39].

A number of epitope-based vaccines targeting self-antigens are in development targeting e.g. Ang II, GIP, Aβ or GnRH [4]. We have shown in this study that AP205 VLP fused to a self-antigen is able to overcome B-cell unresponsiveness. In addition, the antibodies neutralized their target, GnRH, as shown by testosterone level reduction and testis atrophy. An anti-GnRH vaccine may find application as a therapeutic vaccine for prostate cancer. Several vaccines are already in development for this indication.

Similarly, an influenza-pandemic vaccine with a rapid onset of action is highly desirable. The M2 response we obtained is remarkable, since not only was mortality avoided, but also morbidity greatly reduced. Moreover, a 100% protection was achieved even with the high dose of virus used in the challenge. High antibody titres were already reached by day 14 in all animals, demonstrating the rapid onset of the antibody response when immunizing with AP205 RNA phage VLP. RNA bacteriophage VLPs are produced recombinantly in *E. coli*, achieving high yield at a low cost of goods. An additional advantage of using bacteria rather than eggs or eukaryotic cells for production is speed. These features of the technology are highly relevant to the design and production of vaccines for a pandemic. They are also advantageous for the design of vaccines against emerging pathogens or for biodefense applications.

## Material and Methods

### Cloning of coat protein fusions

Four intermediary plasmids for convenient subcloning of fragments coding for epitopes by cassette mutagenesis were prepared by PCR with pAP283 as template (Cielens et al., unpublished). Plasmids pAP378 and pAP382 were designed to introduce epitopes with a short (GSGG), respectively a long amino acid spacer (GSGTAGGGSGS) at the N-terminus, while plasmids pAP409 and pAP405 were designed for introducing epitopes with a short (GSG), respectively a long amino acid spacer (GTAGGGSG), at the C-terminus of AP205 coat protein. Oligodeoxynucleotide pairs were the following: p2.561 (5′-TGCCATGGGATCCGGAGGGGCAAATAAGCCAATGCAACC-3′) and p1.46 (5′-TGAAGCTTAAGCAGTAGTATCAGACGATACG-3′) for pAP378, p2.589 (5′-TGCCATGGGTTCCGGAACCGCGGGCGGGGGATCCGGTTCGGCAAATAA-GCCAATGCAACC-3′) and p1.46 (5′-TGAAGCTTAAGCAGTAGTATCAGACGATACG-3′) for pAP382, p1.45 (5′-TGTCTAGAATTTTCTGCGCACCCATCCCGG-3′) and p2.587 (5′-TGATGCATCCTCCGGATCCAGCAGTAGTATCAGACGATAC-3′) for pAP409 and p1.45 5′-TGTCTAGAATTTTCTGCGCACCCATCCCGG-3′) and p2.588 (5′-TGATGCATAATCCGGAACCGCCTCCTGCGGTTCCAGCAGTAGTATC-3′) for pAP405. The amplified fragments were subcloned into plasmid pQβ185 (pAP378 and pAP382) [40] or pQβ10 (pAP409 and pAP405) [41]. In pAP378 asparagine 14 is mutated to aspartate. Fragments coding for epitopes to be fused at the N-terminus were cloned as a *Nco*I-*Kpn*2I cassette, while constructs for fusion to the C-terminus were cloned as a *Kpn*2I-*Mph1103*I cassette (Fig. 1). The primer pairs for the plasmids coding an epitope fused to AP205 coat protein N- or C-terminus are described in the supplementary Material and Methods (Text S1).

### Expression and purification of AP205-fusion VLPs

Plasmids were transformed into *E.coli* JM109. A seed culture was prepared by inoculating a single colony into LB medium containing 20 mg/l Ampicillin and growing the culture overnight at 37°C without shaking. For expression, the overnight culture was diluted in M9 medium supplemented with casaminoacids (Difco) and containing 20 mg/l Ampicillin and growth of the culture carried out at 37°C with vigorous aeration for 14–20 hours.

Cells were resuspended in TEN buffer (20 mM Tris-HCl, 5mM EDTA, 150 mM NaCl, pH 7.8) containing 1 mg/ml lysozyme and 0.1% Tween 20, and lysed by three freeze thaw cycles followed by ultrasonication. Clarified lysates were purified by one or more sequential gel filtration steps using CL-4B, CL-2B and Sepharose 6B columns (GE Healthcare). Some VLPs were additionally purified by ultracentrifugation over a CsCl gradient. Concentrations of the purified proteins were determined by the Bradford test. For the AP205-GnRH vaccine, two bands reactive with an anti-GnRH antiserum were observed when using vector pAP405 for expression. This wasn't observed in any other construct. Subcloning into a pKK222 derivative however led to a single band. The immunogenicity of both constructs was equivalent.

### Electron microscopy: Negative staining

Suspensions of VLPs were adsorbed on carbon-formvar coated grids and stained with 1% phosphotungstic acid (pH 6.8). The grids were examined with a JEM 100C electron microscope (Jeol Ltd., Tokyo,Japan) at an accelerating voltage of 80 kV. Photographic records (negatives) were performed on Kodak electron image film and electron micrographs were obtained by scanning of negatives with EPSON 2480 PHOTO scanner.

### Anti-peptide antibody response measured by ELISA

Peptides modified to contain a cysteine at their N- or C-termini, and in some cases additionally a diglycine spacer, were conjugated to ribonuclease A (RNAse) using the cross-linker SPDP (Pierce, Rockford, IL, USA). The resulting conjugate was coated overnight at 4°C on maxisorb plates (Nunc, Denmark), at a concentration of 10 µg/ml. Microtiter plates were blocked with 2% bovine serum albumin in PBS supplemented with 0.05% Tween 20. Binding of the sera was detected with a Horseradish-peroxidase goat anti-mouse IgG conjugate. Binding of VLPs to anti-peptide sera was assessed in an inhibition ELISA assay. Serial VLP dilutions were pre-incubated with anti-peptide sera on blocked microtiter plates. Remaining free antibody was detected by ELISA using an RNAse-peptide conjugate as described above. The sera specific for Ang II were raised using Ang II conjugated at its N-terminus to carrier Qβ.

### Immunization of mice

Animal experiments were conducted according to guidelines set by the Swiss Federal Veterinary Office (BVET) and were approved by the Kantonale Veterinäramt of Zürich. Balb/c mice (n = 3) were immunized subcutaneously on day 0 and 14 with 25 µg proteins of AP418, AP420, AP421 and AP422. The proteins were diluted to a final volume of 200 µl in PBS, and 100 µl were injected in the left and right inguinal region of each animal. Animals were bled on day 14 and 21 for measurement of antibody titre.

### Anti-GnRH immunization

Male C57 BL/6 mice (n = 5) were either immunized subcutaneously with 50 µg of AP205-GnRH on day 0 and 21, or untreated (control animals). Animals were additionally bled on day 28 and 45, and were sacrificed on day 70. Testes were sampled, weighed and testosterone was measured in serum.

### Anti-M2 immunization and influenza challenge

C57BL/6 mice were kept in our animal facility, free of specific pathogens. Influenza Virus PR8 (A/PuertoRico8/34, H1N1) was originally provided by J. Pavlovic, University Zuerich. C57BL/6 mice (n = 6) were immunized on day 0 and 18 with 50 µg of M2-AP205 or AP205. On day 31, mice were infected intranasally (i.n.) with 4×LD50 (4×10^4^ p.f.u./ml) of PR8 virus (H1N1) in endotoxin-free phosphate buffer saline (PBS) under isofluran anaesthesia. Mice were monitored for another 22 days thereafter, when body weight and rectal body temperature were determined daily.

## Supporting Information

Text S1 Supplementary Material and Methods.
(0.04 MB DOC)

Figure S1 Electron micrographs of AP205-D2 VLPs displaying the D2 peptide. The D2 peptide was fused to the C-terminus (AP418 - short linker, AP420 - long linker) or the N-terminus (AP421 - short linker, AP422 - long linker) of AP205 coat protein.
(5.78 MB TIF)

Figure S2 Coomassie-stained LDS-PAGE of AP205-D2 VLPs. AP205 VLPs carrying D2 peptide fused to the C-terminus (AP418 and AP420) or N-terminus (AP421 and AP422) of AP205 coat protein, analyzed by LDS PAGE. Lane 1 is the Marker, lane 2 AP205 VLP conjugated chemically to D2 peptide at an epitope density of 1 peptide per AP205 subunit on average. The band corresponding to one AP205 subunit fused to 1 D2 peptide is marked by a star, the band corresponding to 2 peptides by two stars, and the band corresponding to 3 peptides by 3 stars.
(0.89 MB TIF)

Figure S3 Electron micrographs of chimeric AP205 VLPs displaying longer foreign epitopes. Electron micrographs of AP205 VLPs with Nef55 epitope fused to the C-terminus via long linker (AP459), CXCR4 epitope fused to the N-terminus via short linker (AP543), and M2 epitope fused to the N-terminus via short linker (AP551).
(3.14 MB TIF)
