# Supplementary material for: Versatile Virus-Like Particle Carrier for Epitope Based Vaccines
Source: PLoS One. 2010 Mar 23;5(3):e9809. doi: 10.1371/journal.pone.0009809 (PMC2843720; doi:10.1371/journal.pone.0009809)
Supplement: Text S1 — Supplementary Material and Methods. (0.04 MB DOC) [file pone.0009809.s001.doc]

**Supplementary Material and Methods S5**

*Cloning of coat protein fusions*

Oligodeoxynucleotide pairs were the following: p2.561 (5’-TGCCATGGGATCCGGAGGGGCAAATAAGCCAATGCAACC-3’) and p1.46 (5’-TGAAGCTTAAGCAGTAGTATCAGACGATACG-3’) for pAP378, p2.589 (5’-TGCCATGGGTTCCGGAACCGCGGGCGGGGGATCCGGTTCGGCAAATAA-GCCAATGCAACC-3’) and p1.46 (5’-TGAAGCTTAAGCAGTAGTATCAGACGATACG-3’) for pAP382, p1.45 (5’-TGTCTAGAATTTTCTGCGCACCCATCCCGG-3’) and p2.587 (5’-TGATGCATCCTCCGGATCCAGCAGTAGTATCAGACGATAC-3’) for pAP409 and p1.45 5’-TGTCTAGAATTTTCTGCGCACCCATCCCGG-3’) and p2.588 (5’-TGATGCATAATCCGGAACCGCCTCCTGCGGTTCCAGCAGTAGTATC-3’) for pAP405.

The primer pairs for the plasmids coding an epitope fused to AP205 coat protein N- or C-terminus were as follows. Angiotensin II (DRVYIHPF) to the N-terminus: oligo3.218 (5’-TCCCATGGGAGATCGTGTATACATCCATCCATTCGGATCCGGAAC-3’)

and oligo3.219 (5’-GTTCCGGATCCGAATGGATGGATGTATACACGATCTCCCATGGGA-3’); to the C-terminus: oligo3.216 (5’-GATCCGGAGATCGTGTATACATCCATCCATTCTAATGCATTG-3’)

and oligo3.217 (5’-CAATGCATTAGAATGGATGGATGTATACACGATCTCCGGATC-3’). D2 peptide (TSNGSNPSTSYGFAN) to the N-terminus: oligo2.590 (5’-CTCCATGGGAACTTCCAACGGAAGCAATCCGAGCACTTCGTACGGTTTCGCGAATGGATCCGGATC-3’), and oligo2.591 (5’-GATCCGGATCCATTCGCGAAACCGTACGAAGTGCTCGGATTGC -TTCCGTTGGAAGTTCCCATGGAG-3’); D2 peptide to the C-terminus: oligo2.196 (5’-CCTCCGGAACTTCCAACGGAAGCAATCCGAGCACTTCGTACGGTTTCGCGAATTAATGCATCG-3’) and oligo2.197 (5’-CGATGCATTAATTCGCGAAACCGTACGAAGTGCTCGGATTGCTTCCGTTGGAAGTTCCGGAGG-3’). GnRH (EHWSYGLRPG) to the C-terminus: oligo 4.56 (5’-GTTCCGGAGAACACTGGTCCTATGGACTCAGGCCTGGTTAATGCATTG-3’) and

oligo 4.57 (5’-CAATGCATTAACCAGGCCTGAGTCCATAGGACCAGTGTTCTCCGGAAC-3’). N-terminus of CXCR4 (MEGISIYTSD NYTEEMGSGD YDSHKEPSFR EENANFNKI) to the N-terminus: oligo 4-I (5’-CATGGAAGGAATTTCCATATATACTTCGGACAACTACACCGAGGAAATGGGTAGCGGCGACTACGACAGCATGAAAGAACCATCCTTCCGCGAGGAGAATGCAAATTTTAATAAAATTT-3’), and oligo 4-II (5’-CCGGAAATTTTATTAAAATTTGCATTCTCCTCGCGGAAGGATGGTTCTTTCATGCTGTCGTAGTCGCCGCTACCCATTTCCTCGGTGTAGTTGTCCGAAGTATATATGGAAATTCCTTC-3’).

The DNA coding the Nef55 polyepitope (GVGFPVRPQVPLRPMTYKAAVDLSHFLKEKGGLEGPGIRYPLTFGWCFKLVPVEP) was assembled from two fragments generated by PCR amplified from a cDNA encoding a 74 amino acid consensus polyepitope from the HIV Nef protein (Nef74, generous gift of Dr. Adrian Huber). The 5’ fragment was created using oligo p3.242 (5’-GATCCGGAGGTGTGGGTTTCCCGGTTCG-3’) and oligo p3.223 (5’-GGATAACGGATACCTGGACCTTCCAGGCCACCCTTTTG-3’). The 3’ fragment was created with oligo p3.222 (5’-GAAAAGGGTGGCCTGGAAGGTCCAGGTATCCGTTATCC-3’) and oligo p3.225 (5’-CAATGCATTACGGTTCAACCGGCAC-3’).The two resulting fragments were annealed and amplified by assembly PCR using oligos p3.242 and p3.225. The resulting product was digested with *Kpn2I* and *Mph1103I* and cloned in the same restriction sites into the vectors pAP409-44.

N-terminal ectodomain (SLLTEVETPIRNEWGCRCNDSSDG) of Influenza virus M2 protein with an exchange of original P24 for G24 was added to the N-terminus: oligo M2-I (5’-

GGCCATGGGATCTCTGCTGACCGAAGTTGAAACCCCGATTCGTAATGAATGGGGTTGCCGTTGCAATGATTCTTCTGATGGTTCCGGAGG-3’) and oligo M2-II (5’-CCTCCGGAACCATCAGAAGAATCATTGCAACGGCAACCCCATTCATTACGAATCGGGGTTTCAACTTCGGTCAGCAGAGATCCCATGGCC-3’) and to the C-terminus: oligo pINC-14 (5’-

CCGGATCTCTGCTGACCGAAGTTGAAACCCCGATTCGTAATGAATGGGGTTGCCGTTGCAATGATTCTTCTGATGGTTAATGCA-3’) and oligo pINC-15 (5’-TTAACCATCAGAAGAATCATTGCAACGGCAACCCCATTCATTACGAATCGGGGTTTCAACTTCGGTCAGCAGAGAT-3’).
