# Supplementary figures and images for: Versatile Virus-Like Particle Carrier for Epitope Based Vaccines
Source: PLoS One. 2010 Mar 23;5(3):e9809. doi: 10.1371/journal.pone.0009809 (PMC2843720; doi:10.1371/journal.pone.0009809)

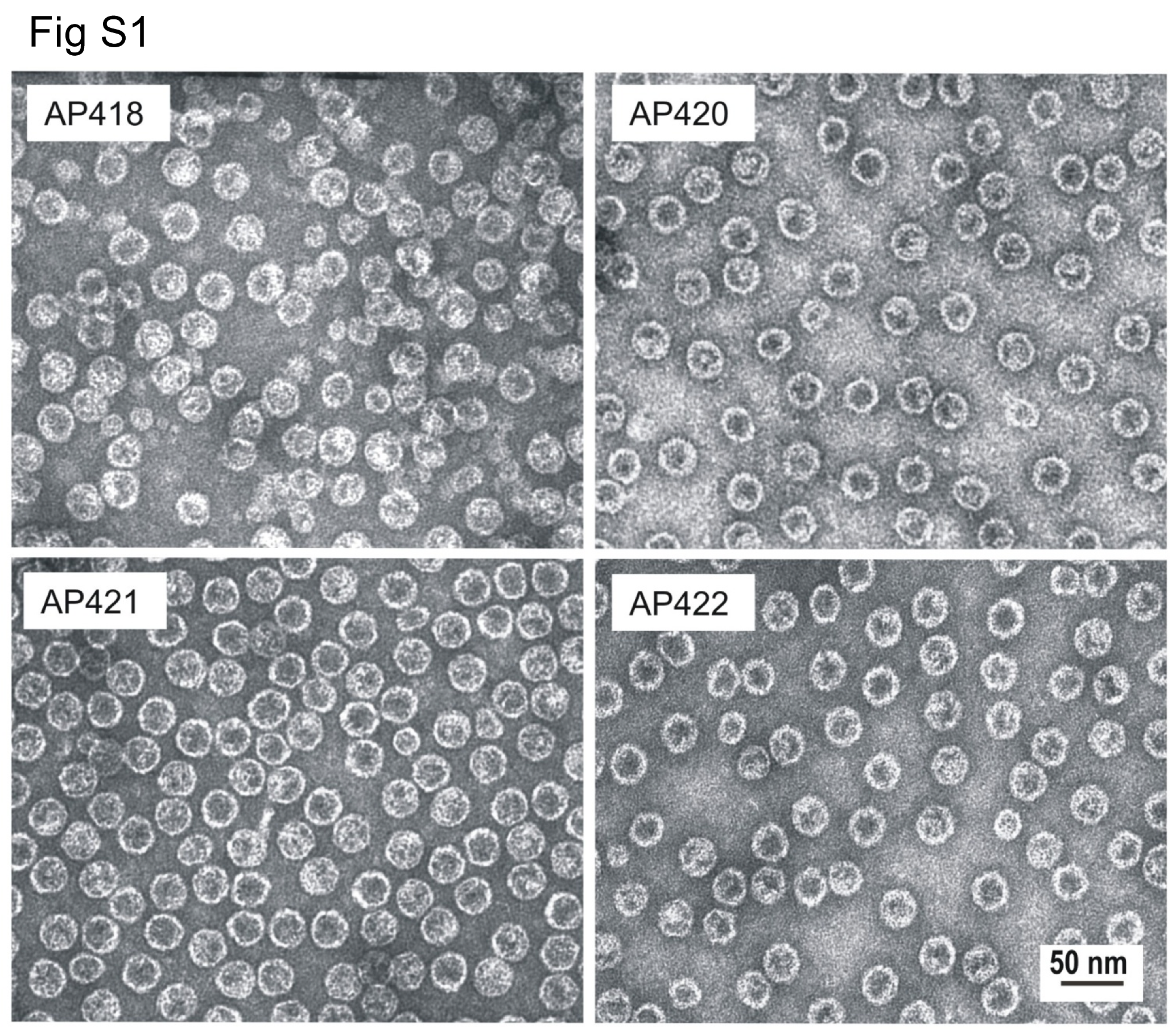

Supplement: Figure S1 — Electron micrographs of AP205-D2 VLPs displaying the D2 peptide. The D2 peptide was fused to the C-terminus (AP418 - short linker, AP420 - long linker) or the N-terminus (AP421 - short linker, AP422 - long linker) of AP205 coat protein. (5.78 MB TIF) [file pone.0009809.s002.tif]

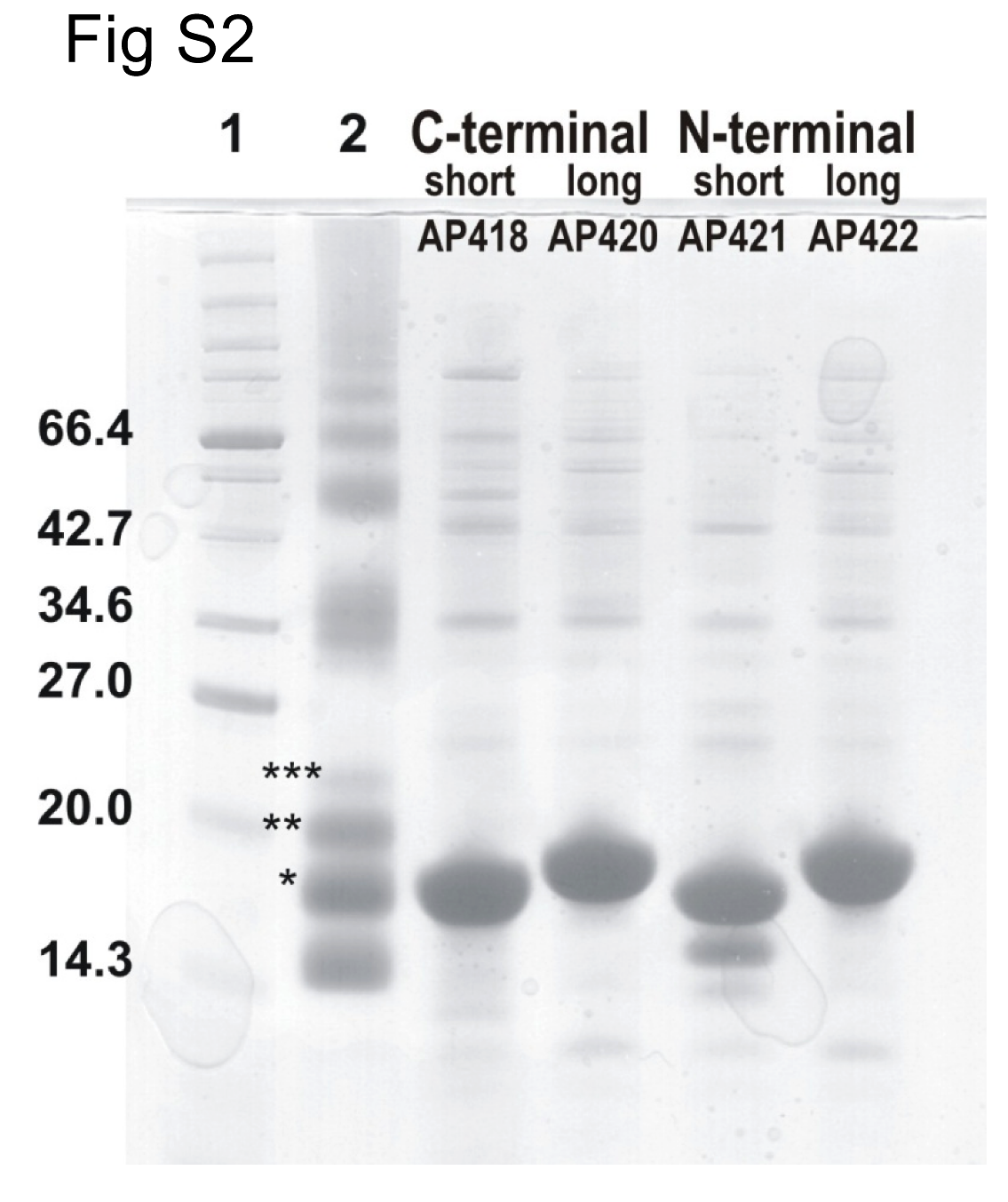

Supplement: Figure S2 — Coomassie-stained LDS-PAGE of AP205-D2 VLPs. AP205 VLPs carrying D2 peptide fused to the C-terminus (AP418 and AP420) or N-terminus (AP421 and AP422) of AP205 coat protein, analyzed by LDS PAGE. Lane 1 is the Marker, lane 2 AP205 VLP conjugated chemically to D2 peptide at an epitope density of 1 peptide per AP205 subunit on average. The band corresponding to one AP205 subunit fused to 1 D2 peptide is marked by a star, the band corresponding to 2 peptides by two stars, and the band corresponding to 3 peptides by 3 stars. (0.89 MB TIF) [file pone.0009809.s003.tif]

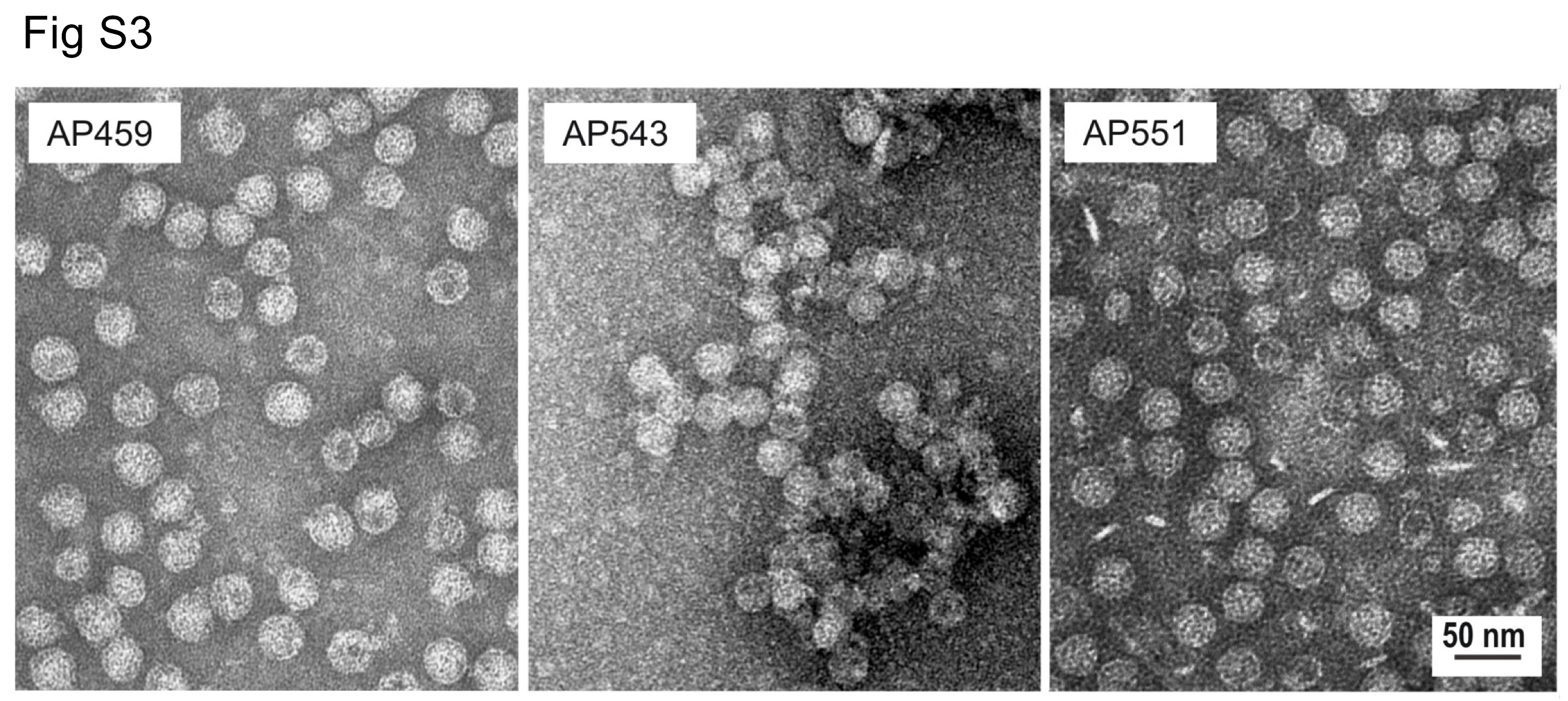

Supplement: Figure S3 — Electron micrographs of chimeric AP205 VLPs displaying longer foreign epitopes. Electron micrographs of AP205 VLPs with Nef55 epitope fused to the C-terminus via long linker (AP459), CXCR4 epitope fused to the N-terminus via short linker (AP543), and M2 epitope fused to the N-terminus via short linker (AP551). (3.14 MB TIF) [file pone.0009809.s004.tif]
